# Precision Horticulture: Application of Optical Sensor Technology for Nitrogen Monitoring Status in Cocoplum, a Native Landscaping Plant

**DOI:** 10.3390/plants12040760

**Published:** 2023-02-08

**Authors:** Bárbara Nogueira Souza Costa, Daniel A. Tucker, Amir Ali Khoddamzadeh

**Affiliations:** Agroecology Program, Department of Earth and Environment, Institute of Environment, Florida International University, Miami, FL 33199, USA

**Keywords:** precision horticulture, environmental horticulture, best management practices, water pollution, fertilization

## Abstract

Cocoplum (*Chrysobalanus icaco*) is an ecologically significant native species to Southern Florida. Application of precision agriculture technologies such as optical sensors reduces the cost of over-fertilization and nutrient runoff. The aim of this work was to establish a base line sensor value for fertilizer treatment in cocoplum by monitoring chlorophyll content using the Soil Plant Analytical Development (SPAD), atLEAF, and Normalized Difference Vegetation Index (NDVI) sensors. Initial slow-released fertilizer treatment 8N-3P-9K was used at 15 g (control), 15 g (supplemented with +15 g × 2; T1), 15 g (+15 g; T2), 30 g (+15 g × 2; T3), 30 g (+15 g; T4), and 45 g (+15 g × 2; T5). Evaluations were conducted at 0 (base reading), 30, 60, 90, 120, 150, and 180 days after treatment. Growth parameters, optical non-destructive chlorophyll meters, leaf and soil total nitrogen and total carbon, and total nitrogen of leachate were analyzed. The results demonstrated that the treatment using 30 g slow-released fertilizer (8N-3P-9K) supplemented twice with 15 g in November and March after the first fertilization in October provided the least contamination through runoff while still providing adequate nutrients for plant growth compared to higher fertilizer concentrations. These results demonstrate that the highest treatment of nitrogen can cause considerable losses of N, causing extra costs to producers and environmental damage due to the flow of nutrients. Thus, techniques that help in N monitoring to avoid the excessive use of nitrogen fertilization are necessary. This study can serve as a basis for future research and for nurseries and farms, since it demonstrated from the monitoring of the chlorophyll content by optical sensors and by foliar and substrate analysis that lower treatments of nitrogen fertilization are sufficient to provide nutrients suitable for the growth of cocoplum plants.

## 1. Introduction

The total crop value of floriculture production in the United States topped $4.80 billion in 2020 with Florida representing 24% of the wholesale value for operations. Within the state of Florida, foliage was by far the largest produced category, evaluated to be $520 million in 2020 with garden plants representing the second highest at $245 million [1].

*Chrysobalanus icaco*, colloquially known as cocoplum or paradise plum, is a woody shrub native to South Florida. Cocoplum is comprised of two distinct ecotypes, inland, and coastal, differentiated by growing conditions and growth habits. Cocoplum is both ecologically and economically significant to South Florida [2].

Nitrogen (N) is one of the most influential macronutrients and is crucial in plant development especially in landscaping plants, being a limiting element of production. Due to this characteristic, it is intensively used in productive crops, aiming to get the crop to reach its maximum potential [3]. Generally, only a minor part of the N applied is recovered by crops, and the excess N is susceptible to loss to the environment where it is associated with various environmental problems. As a native plant, cocoplum’s nutritional requirement is low [2], but as one of the most common landscaping plants in South Florida, it is a frequent practice to fertilize young plants to help with the establishment and expedite the growth. Excess applied N can be leached below the root zone or lost in runoff [4,5,6]. The protection of the environment has become a necessary consideration for intensive agriculture and horticulture. Miami-Dade County in southeastern Florida has enacted a fertilizer ban during the rainy season from 15 May to 31 October aimed at protecting the local waters from fertilizer runoff [7]. Current methods for N fertilizer monitoring are no longer applicable in modern farming due to the long turnover time and the cost of analysis [8,9]. Furthermore, the price of N-based fertilizer has increased from $726 per ton on 15 July 2021, to $1469 per ton on 14 July 2022, an increase of $743 per ton [10]. In addition to the price, the other big challenge is the availability of fertilizer for farmers and nursery producers. A viable alternative method is to use optical sensors for an instant and non-destructive monitoring of the nitrogen status in potted plants.

Good agricultural practices respond mainly to the need of protecting biodiversity, genetic resources and landscape, soil, and water resources, as well as the provision of public goods by farmers. Biodiversity conservation is inextricably linked to agricultural activity [11]. Precision agriculture has the objective of improving agricultural yields and minimizing costs by assisting management with the use of sensors, remote sensing, and information technologies [12]. Precision agriculture has greatly benefited from advances in machine vision and image processing techniques. The use of feature descriptors and detectors allows the finding of distinctive key points in an image, and the use of this approach for agronomical applications has become a widespread field of study [13]. Machine learning in precision agriculture has become a promising approach for increasing productivity without environmental impact [14]. Site-specific nitrogen (N) management in precision agriculture is used to improve nitrogen use efficiency (NUE) at the field scale [15].

The use of visible (VIR) and infrared (NIR) imaging provides information on crop health and growth stage. During the photosynthesis process, chlorophyll molecules of plants absorb blue and red light and reflect green light. On the other hand, infrared light penetrates the inner part of leaves reflecting infrared energy. Since leaf spectral reflectance changes with plants growing, affection of diseases and pest infections, employing these images allows adequate monitoring of crops [16]. Technologies like UAV are implemented to attain information about the crop state in a fast and efficient way. To achieve this task, they employ RGB and multispectral cameras. Reflectance bands provide information on leaf structure, chlorophyll content, and nutritional and water stress, which is useful for determining crop health and subsequent yield enhancement [17].

Several sensors have been designed to measure either the reflectance or the absorbance of green color present in the leaves. The greenness of the leaves represents the amount of chlorophyll found in the chloroplasts, which can be used as an indirect indicator for the photosynthetic processes of the plant to determine plant health and vigor. Growers can use this to monitor plant N levels using sensor readings to determine the nitrogen status of the potted plants [18]. These sensors are referred to as transmittance-based chlorophyll meters. There are currently several commercially available transmittance-based chlorophyll meters, including the Soil Plant Analytical Development (SPAD-502) and the more recent and low-cost atLEAF+ sensor. Reflectance sensors provide information on crop N status by measuring specific wavelengths of radiation absorbed and reflected from crop foliage [19,20,21,22]. Plant tissues normally absorb approximately 90% of the visible radiation (390 to 750 nm) and reflect approximately 50% of the NIR (750 to 1300 nm) [19]. The degree of absorbance and reflectance in the visible and NIR portions of the spectrum varies with crop N content, thus, providing information on the crop N status. To increase the sensitivity to specific biophysical characteristics and reduce variability, spectral vegetation indices that combine spectral reflectance from 2–3 wavelengths are calculated [23,24]. The Normalized Difference Vegetation Index (NDVI) [25] is probably the most widely used, demonstrated by technologies such as the GreenSeeker^TM^, a canopy-wide reflectance sensor.

The aim of this study is to establish a base line sensor value for fertilizer treatment in cocoplum, a woody shrub native to South Florida, by monitoring chlorophyll content using SPAD and atLEAF sensors, and Normalized Difference Vegetation Index (NDVI), by a precision horticulture point of view. At the end of the treatments for six months, growth parameters, total nitrogen in the soil and in the leaf, and a leachate sample were also carried out in order to measure the nutrient runoff to find the best management practices among fertilizer treatment. The results of this study are extremely important and can serve as a basis for future research and for nurses and farms for best management practices among fertilizer treatment for cocoplum.

## 2. Results

The Table 1 shows the acronyms, sensors, and measures of each sensor used in this study.

### 2.1. Growth Characteristics Relative Chlorophyll Content (atLEAF), and Normalized Difference Vegetation Index (NDVI)

The growth characteristics, relative chlorophyll content (atLEAF), and NDVI did not differ significantly for the interaction between fertilization rate and evaluation period represented by days after fertilization (DAF). Therefore, these factors were evaluated separately. Fertilizer treatments were not significantly different in plant height and NDVI. However, atLEAF values and number of leaves were significantly different (*p* ≤ 0.05). The atLEAF values (66.22) and leaves number (215.09) were significantly (*p* ≤ 0.05) higher with using 45 g supplemented with +15 g (November and March; T5) compared to 15 g (control) with 61.97 and 182.91, respectfully (Table 2).

The highest leaf number (227) was recorded after 90 DAF which was significantly (*p* ≤ 0.05) higher, compared to 0, 30, and 60 DAF (107.20, 171.53, and 187.10), respectively. The highest plant height was recorded at 180 DAF with 55.73 cm. These results show the plant growth and the increase in the relative chlorophyll content (atLEAF) and NDVI over the months, during six months of evaluation, showing the normal growth behavior of the plants (Table 3).

Relative chlorophyll content (atLEAF) was significantly higher at 90 DAF (66.64) compared to 0, 30, 60, and 120 DAF (59.43, 61.85, 64.60, and 64.90), respectively. NDVI values were significantly (*p* ≤ 0.05) higher (0.87) in 120 DAF compared to 0, 30, 60, 90, 150, and 180 DAF (0.79, 0.81, 0.83, 0.83, 0.84, and 0.81), respectively. Also, 150 DAF provided a higher value (0.84) for NDVI than 0, 30, and 180 DAF (0.79, 0.81, and 0.81), respectively. Finally, 60 DAF provided a higher (0.81) value for the same feature compared to 0 and 30 DAF (0.79, and 0.81), respectively (Table 3).

### 2.2. Relative Chlorophyll Content (SPAD)

**Figure 1:**
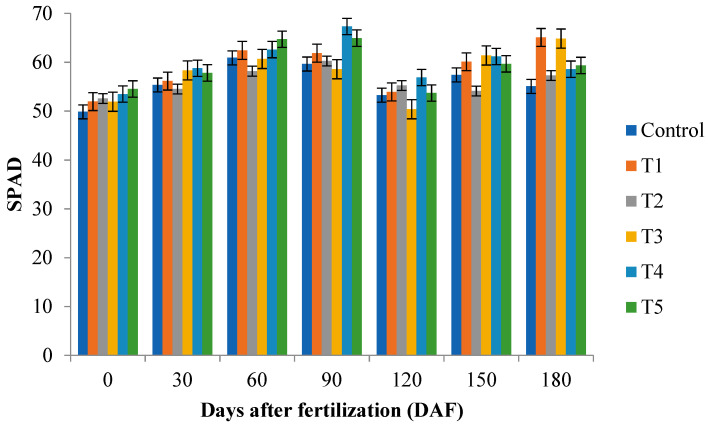
Relative chlorophyll content (SPAD) of cocoplum plants grown in different fertilization rate at 0, 30, 60, 90, 120, 150, and 180 days after fertilization. 15 g (control), 15 g (supplemented with +15 g applied 2 times in November and March; T1), 15 g (+15 g November; T2), 30 g (+15 g November and March; T3), 30 g (+15 g November; T4), and 45 g (+15 g November and March; T5).

There was significant interaction (*p* ≤ 0.05) between fertilization rate and days after fertilization for relative chlorophyll content (SPAD). An increase in relative chlorophyll content (SPAD) (67.32) was observed in the treatment 30 g (+15 g November; T4) at 90 days after fertilization (Figure 1) by Tukey’s test (*p* ≤ 0.05).

### 2.3. Total Nitrogen (TN) and Total Carbon (TC) of Leaf and Substrate Samples

**Figure 2:**
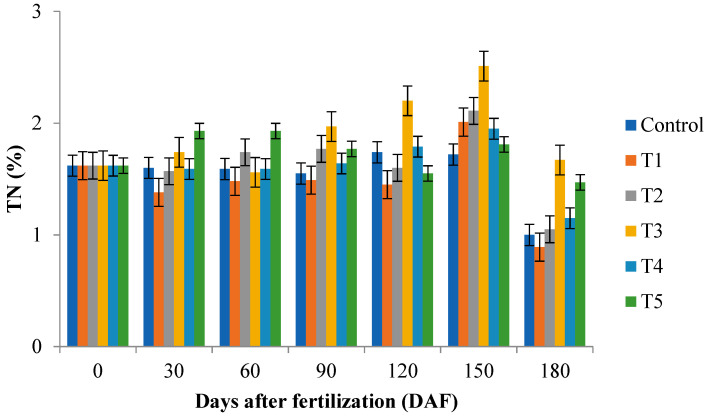

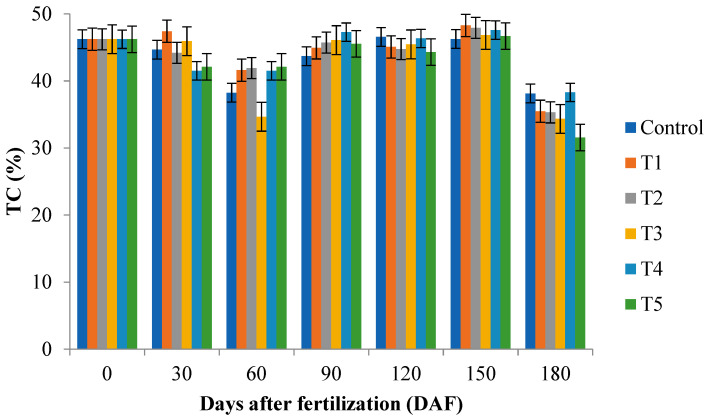
Total nitrogen (TN) and total carbon (TC) of leaf samples of cocoplum plants grown in different fertilization rate at 0, 30, 60, 90, 120, 150, and 180 days after fertilization. 15 g (control), 15 g (supplemented with +15 g applied 2 times in November and March; T1), 15 g (+15 g November; T2), 30 g (+15 g November and March; T3), 30 g (+15 g November; T4), and 45 g (+15 g November and March; T5).

There was significant interaction (*p* ≤ 0.05) between fertilization rate and days after fertilization for total nitrogen and total carbon of leaf samples. An increase in total nitrogen (2.51) was observed using 30 g (+15 g November and March; T3) at 150 days after fertilization, and an increase in total carbon (48.27) was observed in the 15 g (+15 g November and March; T1) at 150 days after fertilization (Figure 2) by Tukey’s test (*p* ≤ 0.05).

There was a significant (*p* ≤ 0.05) interaction between fertilization rate and days after fertilization, contributing to an increase in total nitrogen and total carbon of soil samples in 180 days after fertilization. An increase in total nitrogen (1.67) was observed using 30 g (+15 g November and March; T3) at 180 days after fertilization, while using 30 g (+15 g November; T4) provided an increase (38.29) in total carbon at 180 days after fertilization (Table 4). The treatment 30 g (+15 g November and March; T3) provided higher values of total nitrogen in the leaf (2.51) and in the substrate (1.67). Nitrogen is one of the most influential nutrients of plant development, being a limiting element of production [3].

### 2.4. Salt, Electric Conductivity (EC), and Total Nitrogen (TN) of Leachate Samples

There was a significant (*p* ≤ 0.05) interaction between fertilization rate and days after fertilization for salt, electric conductivity, pH, and total nitrogen. For leachate samples at days 60, 90, 150, and 180 DAF, the treatment 45 g (+15 g November and March; 5) provided a higher value for salt (1622, 2194, 1966, and 1343), and EC (3047, 4116, 3658, and 2626) compared to 15 g (control) (488.60, 679.60, 448.00, and 386.00) and (998, 1370, 925, and 800), respectively. On day 30 DAF, all treatments provided higher values for salt (2830, 2538, 2576, 2968, and 2990) and EC (5206, 4680, 4742, 5470, and 5460) compared to control treatment (846 and 1654, respectively) (Table 5).

Lowest total nitrogen (8.244 ppm) in leachate samples was recorded at 30 DAF using 30 g (+15 g November and March; T3) compared to other treatments. At 60 and 150 DAF the treatment 45 g (+15 g November and March; 5) provided a higher value of total nitrogen (147.500 ppm; 106.500 ppm, respectively) in leachate samples compared to other treatments except the treatment 15 g (+15 g November; T2) at 60 DAF (115.250 ppm), and treatment 15 g (+15 g November and March; T1) at 150 DAF (124.470 ppm) (Table 5). At 180 DAF using 45 g (+15 g November and March; 5), higher values of total nitrogen (46.106 ppm) were recorded in leachate samples compared to 15 g (control) (1.484 ppm), 15 g (+15 g November; T2) (2.362 ppm), and 30 g (+15 g November; T4) (2.845 ppm) (Table 5).

These results demonstrate that the highest treatment of nitrogen can cause considerable losses of N, causing extra costs to producers and environmental damage due to the flow of nutrients. Thus, techniques that help in N monitoring to avoid the excessive use of nitrogen fertilization are necessary.

**Figure 3:**
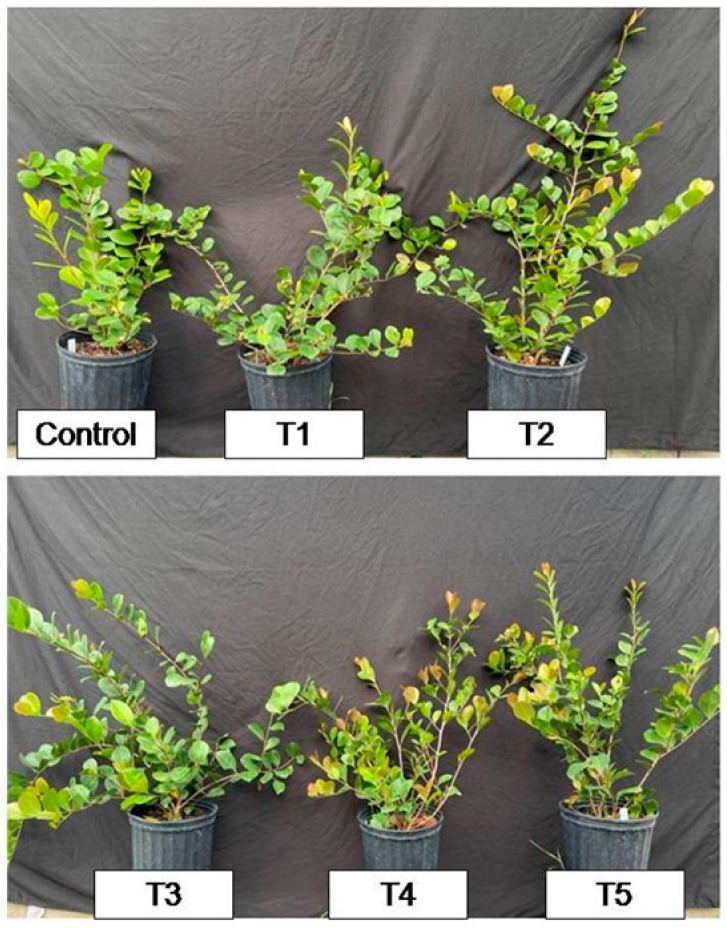
Cocoplum grown in different fertilization rate at the end of the experiment at six months (180 days after fertilization). 15 g (control), 15 g (supplemented with +15 g applied 2 times in November and March; T1), 15 g (+15 g November; T2), 30 g (+15 g November and March; T3), 30 g (+15 g November; T4), and 45 g (+15 g November and March; T5).

This study can serve as a basis for future research and for nurseries and farms, since it demonstrated from the monitoring of the chlorophyll content by optical sensors and by foliar and substrate analysis that lower treatments of nitrogen fertilization are sufficient to provide nutrients suitable for the growth of cocoplum plants (Figure 3). In addition, by providing less contamination by runoff, environmental hazard is avoided

### 2.5. Correlation Coefficient between Sensor Parameters, Number of Leaves (NL), and Total Nitrogen (TN) and Total Carbon (TC) of Leaf Samples

SPAD and atLEAF values were significantly (*p* ≤ 0.05) correlated at 90 DAF. Also, NDVI values were significantly (*p* ≤ 0.05) correlated with NL at 150 DAF. There were not significant correlations observed for any parameters at 30 and 180 DAF (Table 6).

The correlation analysis evidenced a negative significant and high correlation (−0.857) between NDVI and number of leaves at 60 DAF. Also, negative significant and high correlations (−0.849, and −0.811) between NDVI and atLEAF, and total carbon and number of leaves, respectively, at 120 DAF, were observed (Table 6).

## 3. Discussion

In a study conducted by Freidenreich et al. [26], application of precise amount of fertilizer at the right time is the most crucial task for horticultural nursery producers/managers. Therefore, this study used optical sensor reading and plant growth parameters to determine the sustainable ideal fertilizer rate for cocoplum plants as a base guideline in nursery production; furthermore, information collected can be used to determine if SPAD, atLEAF, and GreenSeeker^TM^ are appropriate devices to estimate fertilizer need of the potted plants.

Nitrogen is an essential element for plant growth and development. It is a major component of chlorophyll in plant leaves. Several sensors have been designed to measure either the reflectance or the absorbance of the green color present in the leaves. The greenness of the leaves represents the amount of chlorophyll found in the chloroplasts, which can be used as an indirect indicator for the photosynthetic processes of the plant to determine plant health and vigor [18]. In fact, portable sensors have opened a new approach to acquire crop growth information rapidly and in a non-invasive manner [27].

In this study, the highest fertilizer concentration showed a higher number of leaves when compared to the control; also, the values of relative chlorophyll content (atLEAF) increased with higher fertilizer concentrations, showing the relationship between plant growth and N status. Similar results to this study were reported; Khoddamzadeh and Dunn [28] observed higher values for atLEAF in the highest fertilizer concentration of 15 g and 20 g N treatments in *Chrysanthemum*. In another study, Dunn et al. [29] reported that atLEAF readings increased with increasing N content in *Salvia*, and Swearengin et al. [30] observed that the atLEAF values increased with greater N rates in ‘Helene Von Stein’.

Chlorophyll is the most important pigment of the leaf and one of the most important of the plant since it is through it that plants manage to capture sunlight and use it as an energy source. By means of sensors it is possible to estimate the amount of chlorophyll in the leaf, and thus be able to evaluate the deficiency of nitrogen in the plant, indicating the necessity of nitrogen fertilizer [31].

The highest value for the relative chlorophyll content (SPAD) was provided by treatment 30 g (+15 g November; T4) at 90 DAF (67.32). Other authors observed SPAD readings increased with increasing N content, such as Dunn et al. [32] in *Gaillardia,* Khoddamzadeh and Dunn [28] in *Chrysanthemum*, Dunn et al. [29] in *Salvia,* and Swearengin et al. [30] in ‘Helene Von Stein’. However, Freidenreich et al. [26] observed a higher SPAD value for the 20 g fertilizer rate at eight weeks after a top-dressed treatment (WAT), compared to the highest fertilizer rate of 30, 40, and 50 g, the control, and the 10 g in *Justicia brandegeana*.

Leaf tissue N analysis refers to the measurement of total N content in leaf blades of the most recently fully expanded leaves. It is a long-established method for monitoring crop N status [33,34]. Although tissue analysis is limited as a N monitoring approach, multi-element tissue analysis is useful for diagnosis of possible nutritional problems [35].

Khoddamzadeh and Dunn [28] reported that the Leaf N increased with increasing fertilizer rates through 38 days after a top-dressed (DAT) pretreatment. The results were different from our findings with usage of 30 g with higher Leaf N compared to the highest concentration of 45 g at 90, 120, 150, and 180 DAF. The results proved N monitoring is very important to avoid over-fertilization and at the same time provide adequate nutrients for plant growth. In addition, these findings could serve as a base guideline for cocoplum fertilization in nurseries and landscapes in South Florida.

Carbon-nitrogen metabolism is the most basic and important nutrient metabolism of plants, and its dynamic changes in the plant directly affect the absorption, transformation of mineral nutrition, formation of protein, and so on [36,37]. Therefore, carbon and nitrogen metabolism and their harmony affect plant growth and development [38].

The correlation analysis demonstrated the association between SPAD and atLEAF sensors at 90 DAF; both sensors can be used to monitor the fertilizer status of the potted cocoplums. Another important observation was the association between NDVI and number of leaves at 150 DAF; this correlation demonstrates that the normalized difference vegetation index is positively related to the number of leaves; that is, when there is a linear increase in one parameter, the same thing occurs for the other parameter. Significant negative correlations were also observed between NDVI and number of leaves at 60 DAF, for NDVI and atLEAF, and between total carbon and number of leaves at 120 DAF. For negative correlations, as there is a linear increase in one parameter, the other parameter decreases.

## 4. Materials and Methods

Cocoplum plants (1 year old) were purchased from Santa Barbara Nursery (Miami, FL, USA) in September 2021. The plants were grown at the FIU Organic Garden shade house located at Florida International University, in Miami, Florida. In October 2021, the initial slow-released fertilizer treatment 8N-3P-9K (Harrell’s^®^) was used at 15 g (control), 15 g (supplemented with +15 g applied 2 times in November and March; T1), 15 g (+15 g November; T2), 30 g (+15 g November and March; T3), 30 g (+15 g November; T4), and 45 g (+15 g November and March; T5), applied on the surface of each pot, and well water was then used during irrigations. The treatments T1, T2, T3, T4, and T5 were supplemented with 15 g after the first fertilizing in October (Table 7).

**Figure 4:**
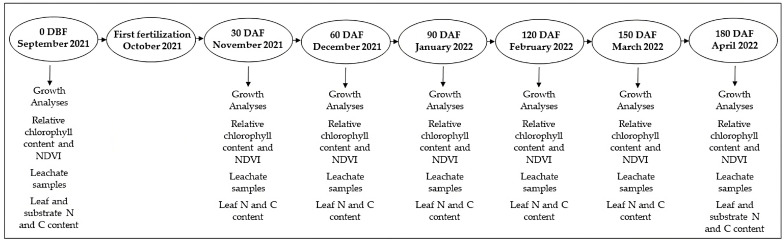
Flowchart with timeline of the sampling. Day before fertilization (DBF)—Base reading. Days after fertilization (DAF).

The evaluations were conducted at 30, 60, 90, 120, 150, and 180 days after fertilization (DAF), and at day 0 (day before fertilization—DBF) as base reading (Figure 4).

The performed analyses were:

### 4.1. Growth Analyses

Five plants per treatment were evaluated monthly to the number of leaves (unit) by a counter, and plant height (cm) by a tape measure. Two branches of each plant, one larger and one smaller, were marked and measured, and the average of these two branches represented the plant height.

### 4.2. Relative Chlorophyll Content and NDVI

**Figure 5:**
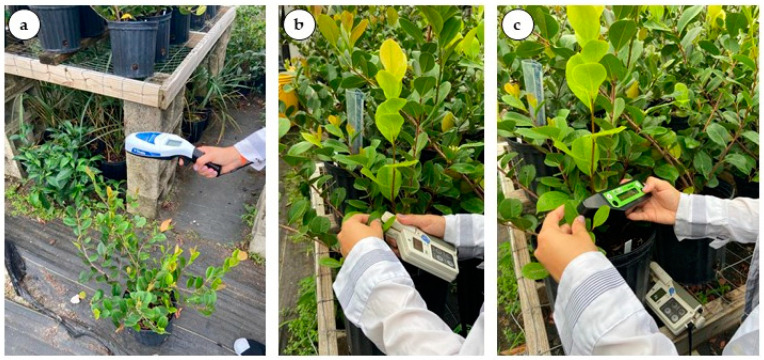
Sensors parameters readings. (**a**) Normalized Difference Vegetation Index (NDVI) by GreenSeeker^TM^. (**b**) Relative chlorophyll content by SPAD. (**c**) Relative chlorophyll content by atLEAF.

Individual plants were scanned from five pots per treatment using a SPAD-502 chlorophyll meter (SPAD-502, Konica Minolta, Japan), an atLEAF chlorophyll meter (FT Green LLC, Wilmington, DE, USA), and a GreenSeeker^TM^ Normalized Difference Vegetation Index (NDVI) sensor (Trimble Agriculture, Sunnyvale, CA, USA). During measurements, the NDVI (Figure 5a) sensor was placed 45 cm above the plant canopy. For the SPAD and atLEAF (Figure 5b,c), measurements were collected from four mature leaves from the middle area of the plant.

### 4.3. Leachate Samples

This analysis was performed from individual containers to determine nutrient runoff rates. Each plant was irrigated until a saturated state was reached. Once containers reached the saturation point, a tray was placed underneath, serving as a collection reservoir. The plants were further irrigated with 350 mL of water, allowing the collection of 50 mL leachate. Samples were stored in 50 mL conical tubes that were immediately refrigerated at 4 °C until laboratory analysis in the CAChE Nutrient Analysis Core Facility at Florida International University; the test was performed for total nitrogen (ppm). The electric conductivity (EC), and salt of each leachate sample was measured in situ.

### 4.4. Leaf and Substrate N and C Content

Five plants per treatment were used for leaf samples and they were collected monthly. For the substrate samples, five plants per treatment were used and they were collected at the beginning and at the end of the experiment. The leaf and substrate samples were dried at 70 °C for 48 h, ground, and then analyzed for the total nitrogen (%) and total carbon (%). These analyses were performed at the CAChE Nutrient Analysis Core Facility at Florida International University.

### 4.5. Statistical Analysis

The experiment was established in a completely randomized design with six treatments that were replicated five times with single pot replications (one plant in each pot), totaling 30 plants. Data were subjected to analysis of variance (ANOVA), and the means were compared by Tukey’s test (*p* ≤ 0.05) using the SISVAR statistical program [39]. The correlation analysis was performed between means of sensor parameters, number of leaves, and total nitrogen and total carbon of leaf samples was performed using the GraphPad Prism version 9.4.1 for Windows, GraphPad Software, San Diego, CA USA, (www.graphpad.com, accessed on 27 July 2022).

## 5. Conclusions

This study was performed by monitoring the chlorophyll content using handheld non-destructive optical sensors to demonstrate a lower amount of fertilizer with providing adequate nutrients for plant health and growth in cocoplum as well as less pollution via runoff, thus reducing environmental damage. The treatment using 30 g slow-released fertilizer (8N-3P-9K) supplemented twice with 15 g in November and March after the first fertilization in October provided the least contamination through runoff while still providing adequate nutrients for plant growth compared to higher fertilizer concentrations. The results of this study could serve as a guideline for nursery producers and landscape personnel as a fast and non-destructive tool for sustainable fertilizer management practices within the ornamental plant industry.

## Figures and Tables

**Table 1:** Acronyms, sensors, and measures of each sensor.

Acronyms	Sensors	Measures
DBF—Day before fertilization	SPAD	Relative chlorophyll content
DAF—Days after fertilization NDVI—Normalized Difference Vegetation Index		
TN—Total nitrogen TC—Total carbon	atLEAF	Relative chlorophyll content
EC—Electric conductivity		
NL—Number of leaves		
SPAD—Soil Plant Analytical Development	GreenSeeker	NDVI

**Table 2:** Number of leaves (NL)**,** plant height, relative chlorophyll content (atLEAF), and normalized difference vegetation index (NDVI) of cocoplum grown in different fertilization rate.

Treatments	NL	Plant Height (cm)	atLEAF	NDVI
Control	182.91 ^b^	47.44 ^a^	61.97 ^c^	0.83 ^a^
T1	189.34 ^b^	45.21 ^a^	64.06 ^b^	0.82 ^a^
T2	195.74 ^ab^	45.42 ^a^	63.55 ^bc^	0.83 ^a^
T3	205.03 ^ab^	47.39 ^a^	63.69 ^bc^	0.82 ^a^
T4	193.83 ^ab^	45.80 ^a^	64.99 ^ab^	0.82 ^a^
T5	215.09 ^a^	45.81 ^a^	66.22 ^a^	0.83 ^a^

Means followed by the same letter within columns are not significantly different by Tukey’s test (*p* ≤ 0.05). 15 g (control), 15 g (supplemented with +15 g applied 2 times in November and March; T1), 15 g (+15 g November; T2), 30 g (+15 g November and March; T3), 30 g (+15 g November; T4) and 45 g (+15 g November and March; T5).

**Table 3:** Number of leaves (NL)**,** plant height, relative chlorophyll content (atLEAF), and normalized difference vegetation index (NDVI) of cocoplum at 0, 30, 60, 90, 120, 150, and 180 days after fertilization.

Days after Fertilization (DAF)	NL	Plant Height (cm)	atLEAF	NDVI
0	107.20 ^c^	33.43 ^e^	59.43 ^d^	0.79 ^e^
30	171.53 ^b^	34.28 ^e^	61.85 ^c^	0.81 ^de^
60	187.10 ^b^	46.90 ^d^	64.60 ^b^	0.83 ^bc^
90	227.07 ^a^	48.97 ^cd^	66.64 ^a^	0.83 ^bcd^
120	238.70 ^a^	51.13 ^bc^	64.90 ^b^	0.87 ^a^
150	223.67 ^a^	52.82 ^ab^	65.68 ^ab^	0.84 ^b^
180	223.67 ^a^	55.73 ^a^	65.45 ^ab^	0.81 ^cde^

Means followed by the same letter within columns are not significantly different by Tukey’s test (*p* ≤ 0.05).

**Table 4:** Total nitrogen (TN), total carbon (TC) of substrate samples of cocoplum plants grown in different fertilization rate at 0 and 180 days after fertilization.

Treatments	Days after Fertilization (DAF)	
	0	180
	TN (%)	
Control	0.85 ^aB^	1.00 ^eA^
T1	0.85 ^aB^	0.89 ^fA^
T2	0.85 ^aB^	1.05 ^dA^
T3	0.85 ^aB^	1.67 ^aA^
T4	0.85 ^aB^	1.15 ^cA^
T5	0.85 ^aB^	1.47 ^bA^
	0	180
	TC (%)	
Control	32.94 ^aB^	38.13 ^bA^
T1	32.94 ^aB^	35.48 ^cA^
T2	32.94 ^aB^	35.31 ^dA^
T3	32.94 ^aB^	34.34 ^eA^
T4	32.94 ^aB^	38.29 ^aA^
T5	32.94 ^aB^	31.57 ^fA^

Means followed by the same letter lower case in the columns (Treatments) and upper case in the rows (DAF) are not significantly different by Tukey’s test (*p* ≤ 0.05). 15 g (control), 15 g (supplemented with +15 g applied 2 times in November and March; T1), 15 g (+15 g November; T2), 30 g (+15 g November and March; T3), 30 g (+15 g November; T4), and 45 g (+15 g November and March; T5).

**Table 5:** Salt, electric conductivity (EC), and total nitrogen (TN) of leachate samples of cocoplum plants grown in different fertilization rate at 0, 30, 60, 90, 120, 150, and 180 days after fertilization.

Treatments	Days after Fertilization (DAF)						
	0	30	60	90	120	150	180
	Salt (ppm)						
Control	277. 60 ^aA^	846.20 ^bA^	488.60 ^cA^	679.60 ^cA^	373.80 ^aA^	448.00 ^bA^	386.00 ^cA^
T1	277. 60 ^aE^	2830.00 ^aA^	897.60 ^bcCDE^	1510.00 ^abBC^	735.60 ^aDE^	2156.00 ^aAB^	1084.80 ^abCD^
T2	277. 60 ^aC^	2538.00 ^aA^	1547.60 ^abB^	1483.80 ^bB^	575.60 ^aC^	619.40 ^bC^	527.40 ^bcC^
T3	277. 60 ^aC^	2576.00 ^aA^	1235.80 ^abcB^	1213.60 ^bcB^	580.00 ^aAB^	2140.00 ^aA^	805.00 ^abcAB^
T4	277. 60 ^aB^	2968.00 ^aA^	1163.80 ^abcAB^	1488.20 ^bB^	942.80 ^aABC^	766.80 ^bAB^	582.00 ^bcAB^
T5	277. 60 ^aE^	2990.00 ^aA^	1622.60 ^aBC^	2194.00 ^aB^	760.40 ^aDE^	1966.00 ^aBC^	1343.60 ^aCD^
	0	30	60	90	120	150	180
	EC (µs)						
Control	581.00 ^a^	1654.60 ^bA^	998.60 ^bA^	1370.00 ^bA^	775.60 ^aA^	925.40 ^bA^	800.60 ^bA^
T1	581.00 ^aE^	5206.00 ^aA^	3789.00 ^aABC^	2868.80 ^abBCD^	1486.20 ^aDE^	4038.00 ^aAB^	2118.20 ^abCDE^
T2	581.00 ^aD^	4680.00 ^aA^	2612.00 ^aAB^	2932.00 ^abBC^	1166.80 ^aBCD^	1137.20 ^bBCD^	1081.00 ^abCD^
T3	581.00 ^aD^	4742.00 ^aA^	2395.20 ^abBC^	2315.00 ^bBCD^	1181.60 ^aCD^	4040.00 ^aAB^	1607.80 ^abCD^
T4	581.00 ^aC^	5470.00 ^aA^	2284.40 ^abBC^	2804.60 ^abB^	2018.40 ^aBC^	1551.80 ^bBC^	1179. 00 ^abBC^
T5	581.00 ^aD^	5460.00 ^aA^	3047.60 ^aBC^	4116.00 ^aAB^	1518.80 ^aCD^	3658.00 ^aB^	2626.80 ^aBC^
	0	30	60	90	120	150	180
	TN (ppm)						
Control	2.903 ^aB^	229.000 ^aA^	8.367 d^B^	4.200 ^aB^	1.300 ^aB^	3.093 ^bB^	1.484 ^bB^
T1	2.903 ^aD^	217.667 ^abA^	53.000 ^cC^	4.300 ^aD^	7.667 ^aD^	124.470 ^aB^	29.764 ^abCD^
T2	2.903 ^aB^	102.74 ^cA^	115.250 ^abA^	5.333 ^aB^	4.633 ^aB^	3.450 ^bB^	2.362 ^bB^
T3	2.903 ^aB^	8.244 d^B^	82.750 ^bcA^	17.167 ^aB^	3.333 ^aB^	15.350 ^bB^	10.838 ^abB^
T4	2.903 ^aC^	175.72 ^bA^	80.750 ^bcB^	5.933 ^aC^	14.867 ^aC^	5.933 ^bC^	2.845 ^bC^
T5	2.903 ^aD^	250.667 ^aA^	147.500 ^aB^	10.167 ^aCD^	6.900 ^aCD^	106.500 ^aB^	46.106 ^aC^

Means followed by the same letter lower case in the columns (Treatments) and upper case in the rows (DAF) are not significantly different by Tukey’s test (*p* ≤ 0.05). 15 g (control), 15 g (supplemented with +15 g applied 2 times in November and March; T1), 15 g (+15 g November; T2), 30 g (+15 g November and March; T3), 30 g (+15 g November; T4), and 45 g (+15 g November and March; T5).

**Table 6:** Correlation coefficient (*r*) for measured sensor parameters, number of leaves (NL), and total nitrogen (TN) and total carbon (TC) of leaf samples in cocoplum at 30, 60, 90, 120, 150, and 180 days after fertilization (DAF).

	atLEAF	NDVI	TN (%)	TC (%)	NL
30 DAF					
SPAD	0.461	−0.048	0.336	−0.613	0.310
atLEAF		−0.394	0.573	−0.120	0.636
NDVI			−0.540	−0.360	0.039
TN (%)				−0.499	0.472
TC (%)					−0.409
60 DAF					
SPAD	0.719	−0.317	0.175	0.066	0.698
atLEAF		−0.055	0.637	0.174	0.490
NDVI			0.693	0.578	−0.857 *
TN (%)				0.409	−0.310
TC (%)					−0.500
90 DAF					
SPAD	0.883 *	0.177	90 DAF	0.694	0.741
atLEAF		0.123	−0.183	0.783	0.769
NDVI			0.073	0.035	0.717
TN (%)			0.432	0.441	0.365
TC (%)					0.624
120 DAF					
SPAD	0.533	−0.637	−0.565	0.123	−0.099
atLEAF		−0.849 *	−0.251	−0.448	0.357
NDVI			−0.023	−0.006	0.180
TN (%)				0.396	−0.454
TC (%)					−0.811 *
150 DAF					
SPAD	0.616	−0.246	0.252	−0.084	−0.132
atLEAF		0.083	−0.134	−0.007	−0.028
NDVI			0.247	−0.399	0.812 *
TN (%)				0.226	0.724
TC (%)					−0.128
180 DAF					
SPAD	0.558	0.666	0.311	−0.316	−0.149
atLEAF		−0.029	0.288	0.295	0.042
NDVI			0.567	−0.369	−0.038
TN (%)				0.426	0.663
TC (%)					0.499

Representing Pearson’s correlation coefficient (*r*) significant at *p* ≤ 0.05 (*).

**Table 7:** Combination of the treatments and fertilizer amounts used. Fertilizer treatments (FT) with supplemented fertilizer treatments (SFT).

Treatments	FT	SFT	Number and Month of Application (SFT)
Control	15 g	----	----
T1	15 g	15 g	2—November and March
T2	15 g	15 g	1—November
T3	30 g	15 g	2—November and March
T4	30 g	15 g	1—November
T5	45 g	15 g	2—November and March

## Data Availability

The dataset is available upon reasonable request to the corresponding author.
